# Development of Quantum Dot (QD) Based Color Converters for Multicolor Display

**DOI:** 10.3390/nano11051089

**Published:** 2021-04-23

**Authors:** Muhammad T. Sajjad, Ashu K. Bansal, Francesco Antolini, Eduard Preis, Lenuta Stroea, Stefano Toffanin, Michele Muccini, Luca Ortolani, Andrea Migliori, Sybille Allard, Ullrich Scherf, Ifor D. W. Samuel

**Affiliations:** 1 Organic Semiconductor Centre, SUPA, School of Physics and Astronomy, University of St Andrews, North Haugh, St Andrews KY16 9SS, UK; ashu.bansal@gmail.com; 2 London Centre for Energy Engineering (LCEE), School of Engineering, London South Bank University, 103 Borough Road, London SE1 0AA, UK; 3 Photonics Micro and Nanostructures Laboratory, ENEA Frascati, Fusion and Technologies for Nuclear Safety and Security Department, Physical Technologies for Safety and Health Division, Via E. Fermi 45, 00044 Frascati, Italy; francesco.antolini@enea.it; 4 Center for Smart Materials & Systems, Bergische Universität Wuppertal, Gauss-Strasse 20, 42119 Wuppertal, Germany; eduard.preis@outlook.de (E.P.); sallard@uni-wuppertal.de (S.A.); scherf@uni-wuppertal.de (U.S.); 5 Petru Poni Institute of Macromolecular Chemistry Gr. Ghica Voda Alley, 41 A, 700487 Iasi, Romania; elenah@icmpp.ro; 6 Istituto per lo Studio dei Materiali Nanostrutturati (ISMN), Consiglio Nazionale delle Ricerche (CNR), Via P. Gobetti, 101, 40129 Bologna, Italy; s.toffanin@bo.ismn.cnr.it (S.T.); mmuccini@bo.ismn.cnr.it (M.M.); 7 Institute for Microelectronics and Microsystems, Consiglio Nazionale delle Ricerche (CNR), Via Gobetti 101, 40129 Bologna, Italy; ortolani@bo.imm.cnr.it (L.O.); migliori@bo.imm.cnr.it (A.M.)

**Keywords:** nanocomposite, energy transfer, narrow emission, thermal annealing, nanocrystal

## Abstract

Many displays involve the use of color conversion layers. QDs are attractive candidates as color converters because of their easy processability, tuneable optical properties, high photoluminescence quantum yield, and good stability. Here, we show that emissive QDs with narrow emission range can be made in-situ in a polymer matrix, with properties useful for color conversion. This was achieved by blending the blue-emitting pyridine based polymer with a cadmium selenide precursor and baking their films at different temperatures. To achieve efficient color conversion, blend ratio and baking temperature/time were varied. We found that thermal decomposition of the precursor leads to highly emissive QDs whose final size and emission can be controlled using baking temperature/time. The formation of the QDs inside the polymer matrix was confirmed through morphological studies using atomic force microscopy (AFM) and transmission electron microscopy (TEM). Hence, our approach provides a cost-effective route to making highly emissive color converters for multi-color displays.

## 1. Introduction

The development of solution-processable materials for color converters has attracted significant attention recently because of their demand in lighting, displays, agriculture, medicine, sensing and communications [1,2,3,4,5,6]. In agriculture, color converters are being used to convert ultraviolet (UV) and violet light into blue and red light to enhance the process of photosynthesis in plants especially at high altitude [2]. In regenerative medicine, they are being used to generate red or near-infrared (NIR) light for deep tissue cancer treatment [3]. In communication and displays, color converters are needed to partially convert the blue light into green and red color [5,7,8]. Among the solution-processable materials, QDs have shown great potential for next-generation displays because of their easy solution-processability, and tuneable optical properties [1,9]. They have been used as color conversion layers in liquid crystal displays (LCDs) as well as in LED (including OLED) displays because of their color purity (narrow emission) and high luminescence efficiency [7,10,11,12]. In LCDs, they have been extensively explored as QD-enhancement films (QDEFs) [7,10,13,14,15] where a mixture of green and red QDs on top of a blue backlight is used to partially convert the blue light into green and red color at the back of the display. This mixture of white light is then used to generate perfectly blue, green and red color using color filters at the front of the displays. Although this approach improved the optical efficiency and color gamut of LCDs, it is inefficient, wasting two-thirds of the light generated. To utilise the efficiency, QDs are being explored as color converters at the front of the display replacing both color filters and QDEFs.

For color conversion, QDs are either deposited on top of blue LEDs to down-convert the blue light to green and red [7] or they are dispersed in photoresist materials and then patterned using photolithography, inkjet printing and e-beam lithography to give a different color [12,16,17]. Recently, QDs were patterned on top of blue OLEDs using inkjet printing and a light conversion efficiency of more than 80% for green-emitting QDs and ~30% for red QDs was achieved [12]. However, the QDs deposition and patterning techniques are not only costly and time-consuming, but also require photomask preparation and rely on the surface modification of films. Therefore, alternate efficient and cost-effective methods need to be explored.

Conjugated polymer-quantum dot (QD) composites have shown great potential as color converters because of their low-cost and unique optoelectronic properties [18,19,20]. To form these composites, usually separate solution of polymers and QDs are prepared and mixed together. In this case, the ligands and surfactants associated with QDs imped the energy transfer, and hence the color conversion properties [21]. We previously proposed an alternative approach of making these composites where luminescent CdSe and CdS QDs were directly grown from the precursor inside a polymer matrix using thermolysis [18] and direct laser patterning [19,22]. There are also other reports where luminescent CdS nanoparticles have been grown in-situ from the thermal decomposition of precursor within a polystyrene matrix [23]. These approaches provide efficient QDs with reasonable light conversion efficiencies, however, the resulting QD emission was very broad. Color purity is one of the main requirements to avoid spectral interference (crosstalk) between different colors in displays. 

In this paper, we developed a process for making highly emissive in-situ QDs with narrow emission inside a polymer matrix. This was done by combining the blue-emitting poly(6-nonylpyridine-2,5-diyl) (Ppy9) polymer with a cadmium-2-(*N*,*N*-dimethylamino) ethylselenolate (CdDMASe) precursor and heating the nanocomposite films at different temperatures under low vacuum. The Ppy9 polymer acted as both an antenna for excitation and an encapsulation shell for QDs. Hence the polymer not only absorbs light and transfers the energy to QDs (due to type 1 energy alignment) but also passivates the surface defects effectively. The photophysical characterisation showed efficient light conversion from blue-emitting polymer to QDs with photoluminescence quantum yield (PLQY) of QDs increased from ~3% in a neat film of QDs to more than 14% in the nanocomposite film of QDs in Ppy9. AFM and TEM confirmed the CdSe nanocrystal formation inside the polymer matrix. 

## 2. Materials and Methods

### 2.1. Materials Synthesis

The protocol for the synthesis of the CdSe precursor cadmium-2-(*N*,*N*-dimethylamino)ethylselenolate (CdDMASe) was adapted from the work of Kedarnath et al. [24]. The synthesis is developed in two steps: the first one involves the formation of an aliphatic diselenide compound and then its reaction with a Cd[II] salt to give the metalorganic precursor. The CdDMASe precursor bearing two Me_2_NCH_2_CH_2_Se fragments, shows good solubility in organic solvents, clean thermal decomposition leading to the generation of cadmium selenide (CdSe) QDs, and is stable at room temperature conditions (can be stored for weeks at 4 °C in the refrigerator).

The monomer and polymer were synthesized according to the literature [25,26]. The polymer was synthesized in a Yamamoto-type coupling reaction and was received with molecular weights M_n_ and M_w_ (GPC in THF) of 70,000 g/mol and 309,000 g/mol, respectively (PDI: 4,4). Thermal stability of the polymer up to 200 °C was proven by thermogravimetry measurement, performed under argon between 35 °C and 700 °C (Supplementary Figure S1).

### 2.2. Thin Film Formation and Photophysical Characterisation 

Thin films of neat precursor, neat Ppy9 and their nanocomposite blends were prepared by spin-coating the solution onto fused silica substrates at 1200 rpm for 60 s. A solution of 20 mg Ppy9 in 1 mL of chloroform was used for spin-coating neat films of Ppy9. Films of neat precursor and nanocomposites (90:10 or 80:20 by precursor:polymer by weight) were prepared by dissolving 50 mg of materials (i.e., mass of precursor plus polymer total 50 mg) in 1 mL of chloroform. The films of neat precursor and nanocomposite blends were baked at temperatures in the range from 140 to 180 °C for 15 min or 30 min under low vacuum (~8 × 10^2^ mbar).

The absorption of films was measured using a Cary 300 UV−Vis spectrophotometer and photoluminescence (PL) spectra were obtained using an Edinburgh Instruments FLS980 fluorimeter. The PL spectrum of the neat precursor without baking was measured using excitation at 380 nm, whereas the PL spectra of neat precursor baked at different temperatures were measured by exciting the films at their corresponding absorption edge. The PL spectra of neat Ppy9 and nanocomposite films were obtained by exciting the films at 330 nm. PLQY of all films was measured using a Hamamatsu integrating sphere C9920−02 luminescence measurement system at similar excitation wavelengths to PL. 

### 2.3. Morphological Measurements Using AFM and TEM

All the film samples were prepared by dissolving the Ppy9 polymer and CdDMASe separately in chloroform at a final concentration of 50 mg/mL. The solution for the film deposition was prepared by mixing the polymer and precursor solutions in the volume ratio of 1:9 (Ppy9:CdDMASe). The samples for AFM were deposited on microscopy glass by spin coating the solution at 1200 rpm for 60 s. AFM topographical images were collected with an NTMDT Solver Scanning Probe Microscope in the tapping mode with a silicon tip. An average value of root-mean-square (rms) roughness was calculated for a scan area of 25 mm × 25 mm.

The samples for TEM characterization were deposited over a TEM grid with spin-coating using the same conditions as for samples deposited on microscopy glass. Copper TEM grids covered by Quantifoils Holey Carbon film were used to maximize the area of the freestanding sample.

Transmission electron microscopy (TEM) measurements were performed with a Philips Tecnai F20 Schottky FEG operating at 200 kV. The QD size was determined manually on the basis of the obtained high-resolution transmission electron microscope (HRTEM) images. The chemical composition was verified using energy dispersive spectroscopy (EDS) with an EDAX Phoenix spectrometer equipped with an ultra-thin window detector and TEM image and analysis software. 

## 3. Results and Discussion

### 3.1. Photophysical Characterisation of Neat Films of Polymer and CdMASe Precursor

**Figure 1:**
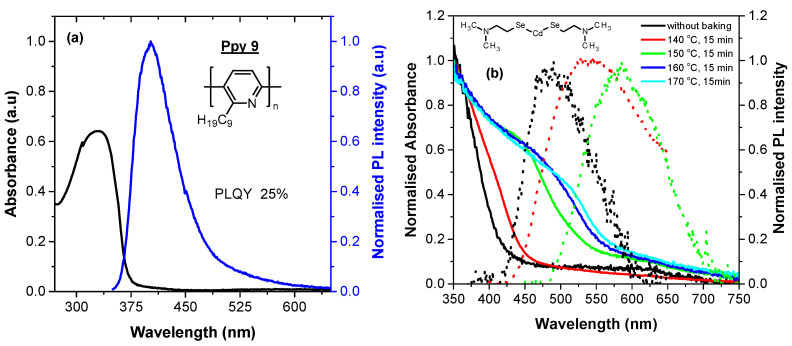
Absorption and fluorescence spectra of neat films. (**a**) poly(6-nonylpyridine-2,5-diyl) (Ppy9); (**b**) CdDMASe precursor before annealing and after annealing at different temperatures for 15 min. The molecular structures of materials are given in the inset.

The absorption and PL spectra of a neat film of Ppy9 are given in Figure 1a. There is an absorption peak around 330 nm and PL emission in the blue region peaking at 403 nm. The PLQY was measured to be 25 ± 3%.

Figure 1b shows the absorption and PL spectra of a neat film of CdDMASe precursor before and after annealing at different temperatures for 15 min. The figure shows that annealing of CdDMASe precursor at ≥150 °C leads to the development of a shoulder in the absorption spectra. This is an indication of conversion of the precursor into QDs. This shoulder shifts towards longer wavelength for higher temperature due to larger nanocrystal size and concentration. The PL follows similar trends to absorption and shifts with temperatures, however, these nanocrystals are not very emissive and also their emission is very broad. The highest PLQY of a film of neat QDs was 3.2 ± 1%. The broad PL emission and low PLQY indicates the presence of either defects (possibly surface defects) or clusters of QDs.

### 3.2. Photophysical Characterisation of Nanocomposite Polymer Films

**Figure 2:**
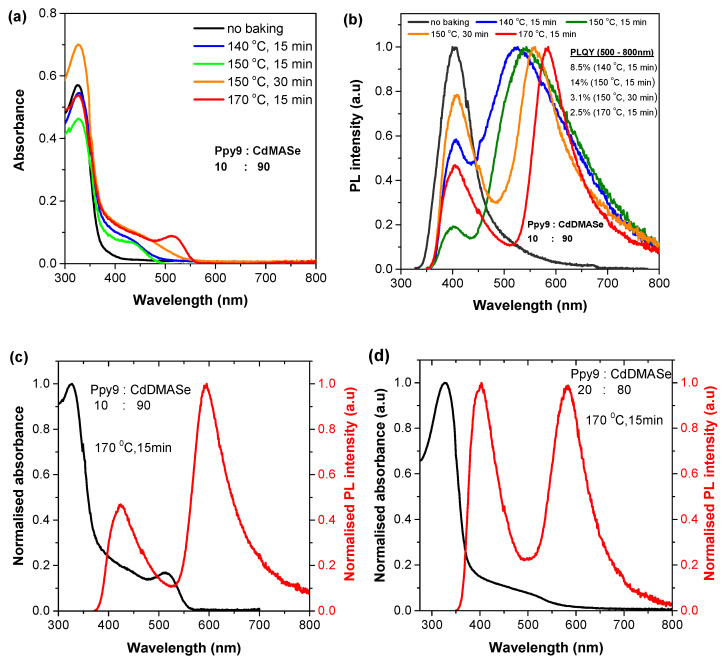
(**a**,**b**) Absorption and PL spectra of Ppy9 and CdDMASe blend film before and after baking at 140 °C for 15 min, 150 °C for 15 min and 30 min, and 170 °C for 15 min. PL spectra were obtained using excitation wavelength of 330 nm in all cases. (**c**,**d**) Absorption and PL spectra of blends of Ppy9 and CdDMASe with different blend ratios. The films were annealed at 170 °C for 15 min.

The absorption spectra of nanocomposite films (with 90% precursor and 10% Ppy9) before and after annealing at temperatures ranging from 140 °C to 170 °C are shown in Figure 2a, and the corresponding measurements for 80% precursor and 20% Ppy9 are shown in the supplementary information (Figure S2). Before annealing, the absorption spectra of nanocomposite films in both cases (80:20, 90:10) have a strong peak around 330 nm due to Ppy9 and a tail beyond 375 nm due to the presence of precursor in the film. After baking at 140 °C, this tail in the case of 90:10 blend turned into a strong shoulder around 435 nm due to formation of QD from the decomposition of precursor. Annealing of 90:10 blends at ≥150 °C leads to a well-defined peak which shifts to longer wavelengths for higher annealing temperature. For instance, for annealing at 150 °C, the absorption spectrum has a peak around 442 nm, whereas for 170 °C, this peak shifted to 517 nm in addition to the Ppy9 peak around 330 nm (Figure 2a). The presence of well-resolved peaks above 430 nm in absorption spectra of nanocomposite films, which red-shift at higher annealing temperatures clearly indicates the formation of QD inside the polymer. These results are different from our previous studies using CdDEX and CdDMASe precursors where a broad shoulder was observed instead of clear peaks due to the polydispersity of QD size in the blend films [18,19,27]. 

**Figure 3:**
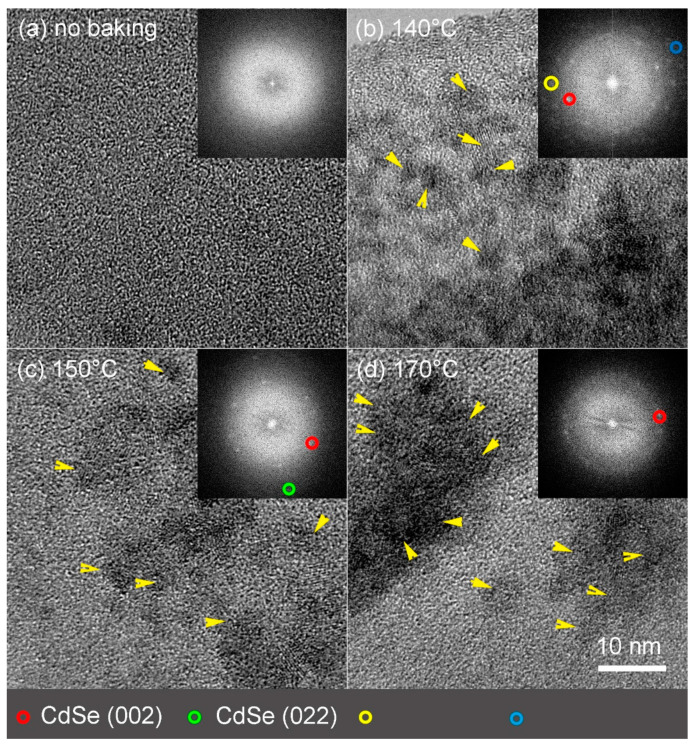
TEM characterization of Ppy9/CdDMASe precursor blends. The yellow arrows indicate the growing QDs. (**a**) High-resolution image of the film, before baking. (**b**) HRTEM of the film baked at 140 °C; (Inset) FFT of the particles highlighting the crystalline reflections (circles). The blue and yellow circles indicated unknown reflections. (**c**) HRTEM of the film baked at 150 °C; (Inset) FFT of the particles highlighting the cubic crystalline structure of the particles (circles). (**d**) HRTEM of the film baked at 170 °C; (Inset) FFT of the particles highlighting the cubic crystalline structure of the particles (circles).

**Figure 4:**
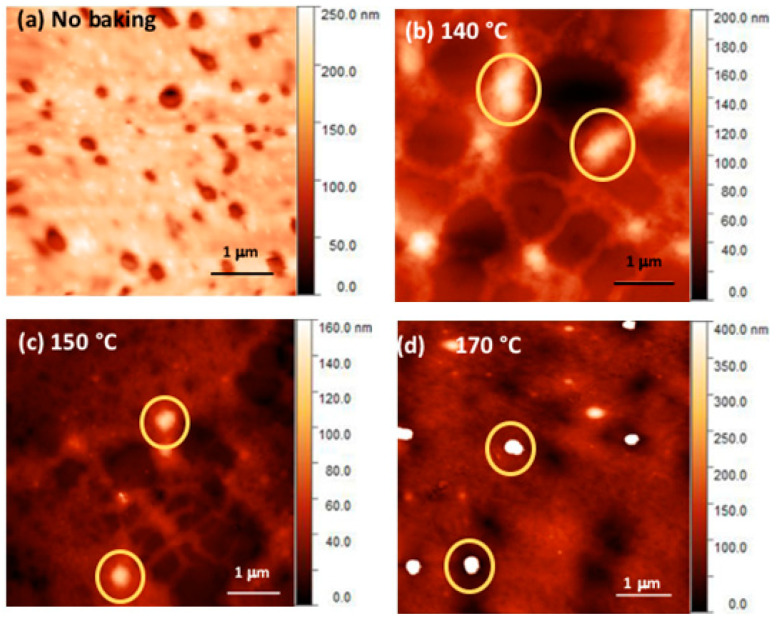
AFM images of blends of Ppy9 and CdDMASe before annealing (**a**) and after annealing at 140 °C for 15 min (**b**) 150 °C for 15 min (**c**) and 170 °C for 15 min (**d**). Large aggregates disappear at higher baking temperatures. The yellow circles highlight the clusters as described in the text. The cross sections of the films are reported in the Supplementary Information.

Figure 2b shows the PL spectra of nanocomposite films with 90:10 blend ratio of precursor/polymer. The PL results for 80:20 blend ratio are given in Supplementary Figure S2b. The PL spectra were obtained by exciting the nanocomposite films at 330 nm (absorption peak of Ppy9). Before baking, the nanocomposite film showed a PL peak around 400 nm which is similar to PL of the neat film of Ppy9 (shown in Figure 1a). This means that precursor is not emissive in the nanocomposite. After baking, the PL spectra showed contributions from both the polymer (at ~400 nm) and nanocrystals (>520 nm). For instance, the film baked at 140 °C for 15 min has an emission peak at 403 nm due to Ppy9 and a broad emission at 525 nm that is not present in the unbaked film. This extra emission has been assigned to the in-situ QDs which formed due to thermal decomposition of the precursor [18]. Increasing the baking temperature/time improved the emission color purity i.e., the emission from QDs becomes narrower. The broad emission at lower baking temperatures (140 °C and 150 °C for 15 min) indicates the formation of very small CdSe nanocrystals in this temperature range. A temperature of 160 °C is required for the full decomposition of the selenolate complex [24]. As the temperature/time increases (170 °C for 15 min and 150 °C for 30 min), narrow emission starts to appear which indicates that QDs acquire more regular structure and there is energy transfer to them from Ppy9. Previously, we observed very broad emission from the same in-situ CdSe QDs in the nanocomposite with blue-emitting polyfluorene [18] or hole transport material (TPBI) [19]. This broad emission was assigned to the presence of a large number of surface defect/trap states as well to a large distribution of QD sizes. However, in the present case, we obtained narrow emission which indicates the presence of small QD size distribution and a very low density of defects state. The QD formation and small size distribution was confirmed by TEM and AFM studies (Figure 3 and Figure 4). At lower resolution, the fluorescence microscopy images show the homogenous color formation with blue emission before baking, and orange emission after baking at 170 ^°^C for 15 min (Figure S3). This is similar to the color we observed in the PL spectra.

To show the effect of annealing more clearly, we plotted the absorption and PL spectra of nanocomposite films for both blend ratios (90:10, 80:20) baked at 170 °C for 15 min in Figure 2c,d. In both cases, the PL spectra show the first evidence of defect-free QD emission [28] for QDs formed inside a conjugated polymer matrix. These results show that the polymer has two functions. The first is that it acts as an antenna that absorbs light and transfers its energy to the QDs. The second is that it helps to control the homogeneity of the QDs and passivate surface defects. This dual function is a major advance compared to previous work [29,30,31] in which commercial QDs were used to achieve narrow emission, whereas in our case, the precursor is used to produce QDs. Our method is efficient and reliable because the precursor is mixed together with polymer and dissolved in a common solvent to achieve a homogenous distribution of QDs. Hence, our results show that thermal annealing converts the precursor into QDs and efficient energy was transferred from polymer to these in-situ formed QDs.

This efficient energy transfer (light conversion efficiency) process was confirmed through PLQY measurements. The samples were excited at 330 nm and PLQY was measured using a Hamamatsu integrating sphere. The PLQY of the polymer in the nanocomposite film before baking was 7.1% which is low compared to the PLQY of the neat film of Ppy9 (25%). This shows that the polymer transfers approximately 70% of its emission to the precursor, however precursor in the nanocomposite is not emissive. After baking, the PLQY of the nanocomposite increases initially with the increase in temperature up to 150 °C and then decreases with further increase in temperature. We extracted the PLQY of QDs by selecting the emission range from 500 nm to 800 nm. We obtained a PLQY of 8.5 ± 0.9 % for heating the films at 140 °C for 15 min, 8.2 ± 0.8% for 140 °C for 30 min, 14% for 150 °C for 15 min, 3.1 ± 0.3 % for 150 °C for 30 min, 2.5 ± 0.3% for 170 °C for 15 min, and <1% for film baked at 180 °C for 15 min. The low PLQY at high temperature may be due to the presence of a higher density of trapping states (e.g., surface-defect states) [19,32]. 

### 3.3. Morphological Studies of Neat Films of Polymer and CdDMASe Precursor

Transmission electron microscopy (TEM) measurements were performed to obtain further information about nanocrystal formation. Figure 3a shows a high-resolution image of the film before baking, where only the amorphous structure of the polymeric film over a hole of the carbon foil of the TEM grid is visible. 

After the baking at 140 °C the HRTEM image shows the appearance of small clusters (yellow arrows), with evidence for short-range atomic ordering typical of early stage crystallites. The Fast Fourier Transform (FFT) of the image (inset of Figure 3b) shows only one (002) reflection (red circle) typical of CdSe cubic structure, with spacing of 0.35 nm, and two unknown reflections probably due to very small growing CdSe crystals [33] (yellow and blue circles). The presence of crystalline nanoparticles together with small unknown structures left in the nanocomposite film baked at 140 °C indicates that this baking temperature is not enough for complete nucleation and growth of the nanoparticles.

Further increases of the baking temperature at 150 °C induce the formation of more defined clusters formed by nanoparticles with a diameter of 4–6 nm (Figure 3c). The FFT shows reflections corresponding to (002) (red circle) and (022) (green circle) from CdSe cubic phase, spaced respectively by 0.35 nm and 0.215 nm. When the film is baked at 170 °C the clusters visible in the HRTEM image (Figure 3d) are formed by approximately 10–30 nanoparticles with size 4–8 nm, with the FFT showing (002) reflections from CdSe cubic phase. Larger view of films also confirmed the QDs and clusters formation as a function of the temperature (Figure S4). 

To study the effect of growing QDs on film surface morphology, Atomic Force Microscopy (AFM) analysis was carried out. Figure 4 shows a typical topographic image of the nanocomposite films of Ppy9 and CdDMASe precursors before and after baking at different temperatures. Before baking, the film shows a homogeneous distribution of holes of about 0.5 μm diameter on the surface (Figure 4a). The presence of the holes is constant at all the observed baking temperatures (Figure S5). The RMS roughness was 25.9 nm. The film baked at 140 °C for 15 min shows the formation of homogeneously dispersed large aggregates of 300–600 nm (yellow circles) covering the film surface with RMS roughness of about 40 nm. In the film baked at 150 °C, some of these large aggregates present at 140 °C decrease their size (200–400 nm) (Figure 4c yellow circles) and the roughness decreases to 17 nm. For the film baked at 170 °C, the aggregates of 200–400 nm size become more defined and appear homogeneously distributed in the film (Figure 4d) (RMS roughness 29.8 nm). The AFM analysis shows that the aggregates start to appear after the baking at 140 °C as the first QDs crystals begin to grow and this is in accordance with the TEM results. However, the size of the aggregates (100–600 nm) is quite different from the QDs size especially at the initial phase of growth. Indeed, at this stage, the precursor decomposes to form the QDs in large areas of the film as shown also by TEM (Figure S4b). The interaction of the growing QDs with the polymer can cause the formation of these large areas of different height as seen in AFM. As the temperature increases, the size of the aggregates decreases (100–400 nm) and they become more defined and this correlates with the formation of better defined QDs as reported by TEM. The aggregates observed by AFM can be formed by several neighboring clusters observed by TEM enveloped within the polymer. 

From these AFM images, it is not possible to get information about single QD, but it can be seen that the aggregates size decreases significantly with increasing the baking temperature. Overall, we found that large aggregates disappear with the increase in temperature and films showed lower RMS roughness at higher temperatures. These results are in correlation with the photophysical results where narrow PL emission was observed at the higher temperatures. 

Both TEM and AFM show the presence of clusters of QDs even if these clusters differ in their size. Indeed, AFM shows a cluster size of hundreds of nanometers, while the TEM displays clusters of around 10 nm. This difference could be due to the polymer that incorporates the clusters and contribute to the enhancement of the observed size with the AFM. In nanocomposites, there is a possibility of both charge and energy transfer, however, efficient energy transfer occurs due to favorable Type 1 energy alignment between the polymer and thermally decomposed QDs (Figure S6).

## 4. Conclusions

In conclusion, we showed that QD based color converters can be made by a solution process method by mixing a CdDMASe precursor with blue-emitting polymer. The in-situ formation of quantum dots occurs when the film is baked at 170 °C for 15 min. We found that the use of a polymer not only acts as an antenna leading to energy transfer to the QD, but also passivates the surface. We found that the size and emission color (and/or purity) of QDs can be controlled by baking temperature/time. Our final QDs showed narrow emission, which is suitable for next generation display technology.

## Data Availability

The research data supporting this publication can be accessed at this article.

## Supplementary Materials

The following are available online at https://www.mdpi.com/article/10.3390/nano11051089/s1, Figure S1: Thermogravimetric analysis of PPy9 performed under argon between 35 °C and 700 °C, showing its thermal stability up to 200 °C; Figure S2. Absorption (a) and PL spectra (b) of blends of Ppy9 (20%) and CdDMASe (80%). The films were annealed at 140 °C, 150 °C and 170 °C for 15 min. PL spectra were obtained using excitation wavelength of 330 nm; Figure S3. Fluorescence images of nanocomposite films of Ppy9 and CdDMASe before (left) and after baking at 170 °C for 15 min (right). These images were captured with a fluorescence microscope using an excitation wavelength of 355 nm; Figure S4. Larger view of the film during the heating process indicating the CdSe QDs distribution within the film under different baking conditions: (a) unbaked film, (b) film at 140 °C with small crystals presence, (c) film at 150 °C with presence of CdSe QDs and initial aggregation process (clusters of QDs); (d) film at 170 °C film with several clusters of QDs; Figure S5. Cross sections of the unbaked (a) and baked films at 140 °C (b), 150 °C (c) and 170 °C (d). In each panel the AFM image of the film (on the left, consider Figure 4 in the manuscript) and the cross-section (right) measured along the line marked 1 in each AFM image is shown. In order to compare the morphology of each film, the depth of voids statistically present on the film surface are also given; Figure S6. Energy levels of polymer Ppy9 and CdSe, showing type 1 alignment.
