# Supplementary material for: Development of Quantum Dot (QD) Based Color Converters for Multicolor Display
Source: Nanomaterials (Basel). 2021 Apr 23;11(5):1089. doi: 10.3390/nano11051089 (PMC8145343; doi:10.3390/nano11051089)
Supplement: Supplementary file 1 [file nanomaterials-11-01089-s001.zip › nanomaterials-1137038-supplementary.pdf]

nanomaterials

MDPI

Supplementary Information

Development of Quantum Dots (QD) Based Color Converters for Multicolor Display

Muhammad T. Sajjad [1,2,*], Ashu K. Bansal [1], Francesco Antolini [3], Eduard Preis [4], Lenuta Stroea [5], Stefano Toffanin [6], Michele Muccini [6], Luca Ortolani [7], Andrea Migliori [7], Sybille Allard [4], Ullrich Scherf [4] and Ifor D. W. Samuel [1,*]

1 Organic Semiconductor Centre, SUPA, School of Physics and Astronomy, University of St Andrews, North Haugh, St Andrews, Fife KY16 9SS, UK; ashu.bansal@gmail.com
2 London Centre for Energy Engineering (LCEE), School of Engineering, London South Bank University, 103 Borough Road, London, SE1 0AA, UK
3 ENEA Frascati, Via E. Fermi 45, 00044 Frascati (Rome); dr.antolini.work@gmail.com
4 Institut für Polymertechnologie Bergische Universität Wuppertal, Gauss-Strasse 20, 42097 Wuppertal, Germany; preis@uni-wuppertal.de (E.P.); sallard@uni-wuppertal.de (S.A.); scherf@uni-wuppertal.de (U.S.)
5 Petru Poni Institute of Macromolecular Chemistry Gr. Ghica Voda Alley, 41 A, 700487 Iasi, Romania; elenah@icmpp.ro
6 Consiglio Nazionale delle Ricerche (CNR) – Istituto per lo Studio dei Materiali Nanostrutturati (ISMN), Via P. Gobetti, 101, Bologna, Italy; s.toffanin@bo.ismn.cnr.it (S.T.); mmuccini@bo.ismn.cnr.it (M.M.)
7 Consiglio Nazionale delle Ricerche (CNR) – Institute for Microelectronics and Microsystems, Via Gobetti 101, Bologna (BO), Italy; ortolani@bo.imm.cnr.it (L.O.); migliori@bo.imm.cnr.it (A.M.)

* **Correspondence:** sajjadt@lsbu.ac.uk (M.T.S.); idws@st-andrews.ac.uk (I.D.W.S.).

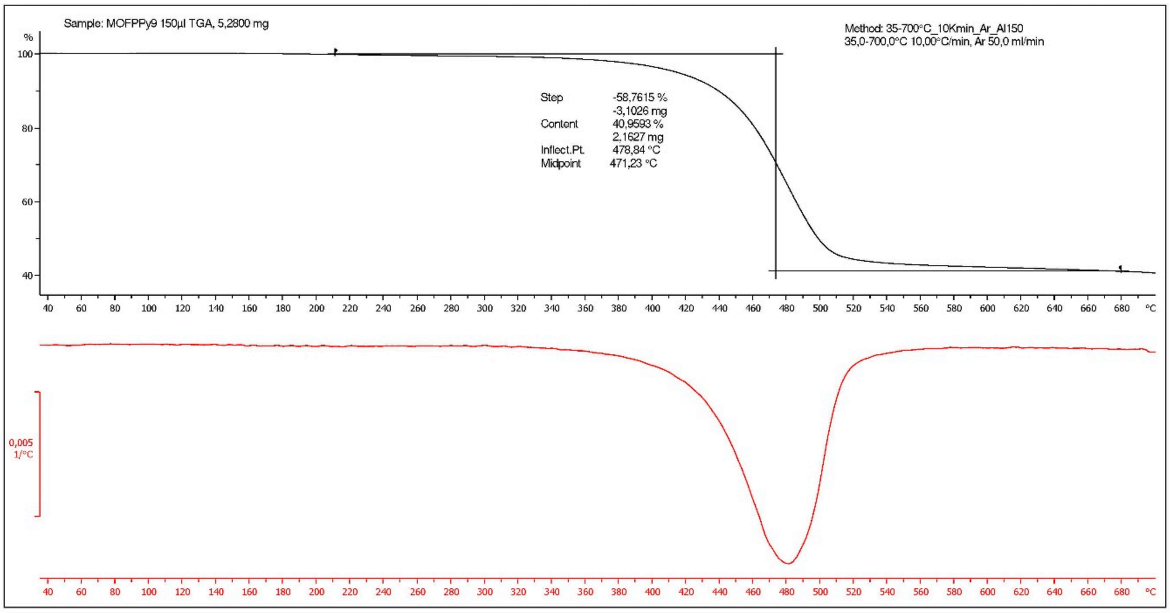

Figure S1. Thermogravimetric analysis of PPy9 performed under argon between 35 °C and 700 °C, showing its thermal stability up to 200 °C.

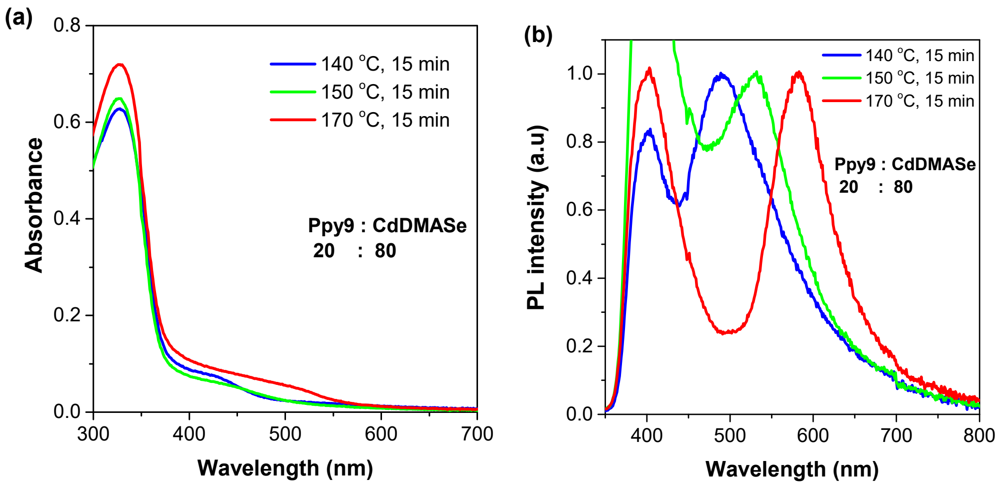

Figure S2. Absorption **(a)** and PL spectra **(b)** of blends of Ppy9 (20%) and CdDMASe (80%). The films were annealed at 140 °C, 150 °C and 170 °C for 15 min. PL spectra were obtained using excitation wavelength of 330 nm.

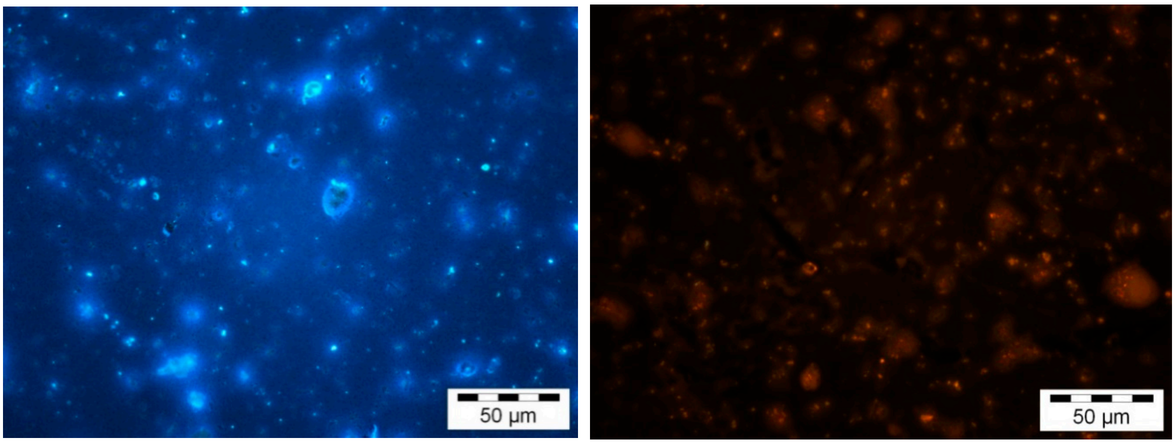

Figure S3. Fluorescence images of nanocomposite films of Ppy9 and CdDMASe before (left) and after baking at 170 °C for 15 min (right). These images were captured with a fluorescence microscope using an excitation wavelength of 355 nm.

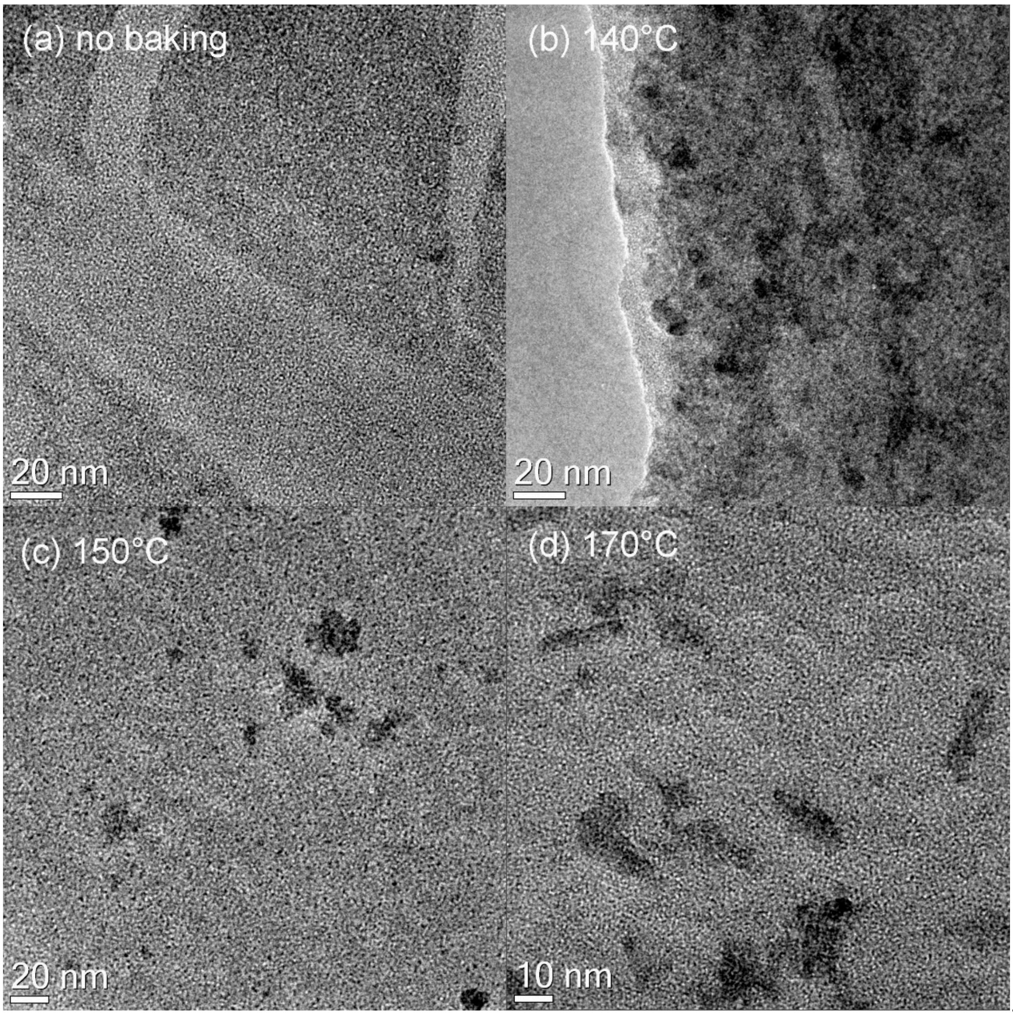

Figure S4. Larger view of the film during the heating process indicating the CdSe QDs distribution within the film under different baking conditions: **(a)** unbaked film, **(b)** film at 140 °C with small crystals presence, **(c)** film at 150 °C with presence of CdSe QDs and initial aggregation process (clusters of QDs); **(d)** film at 170 °C film with several clusters of QDs.

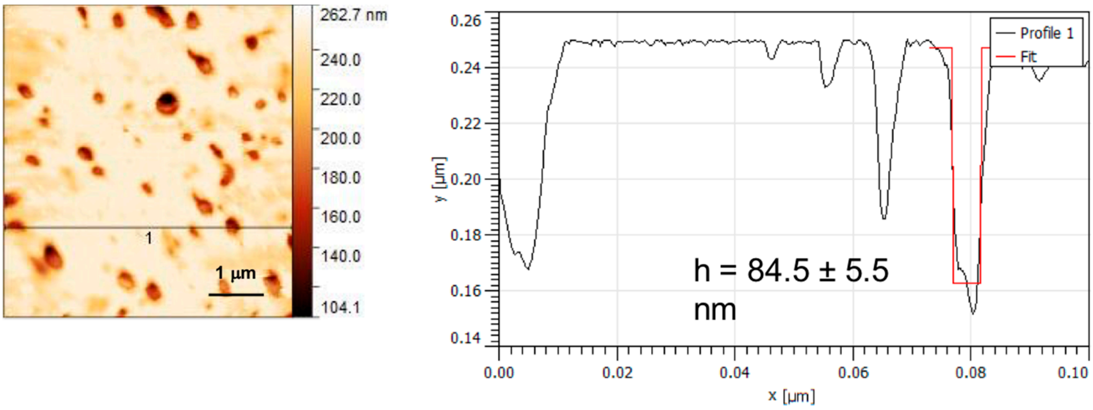

S5a.

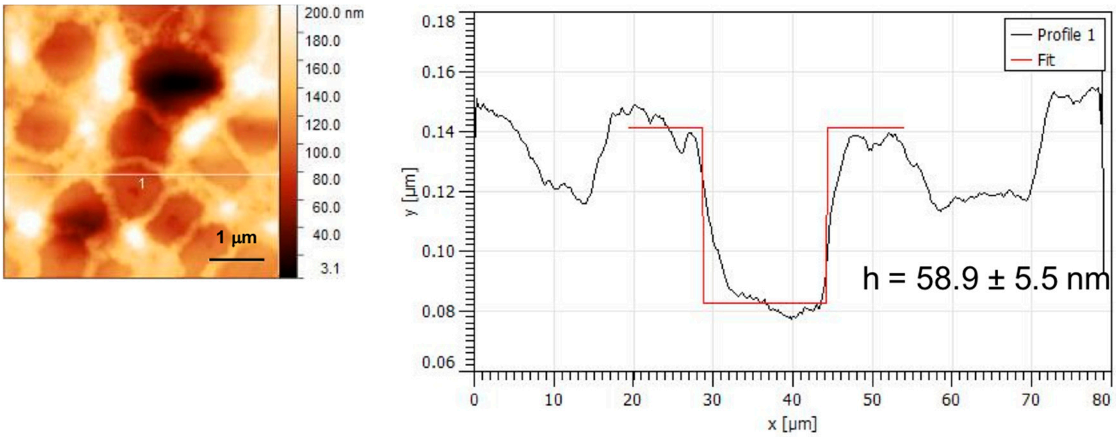

S5b.

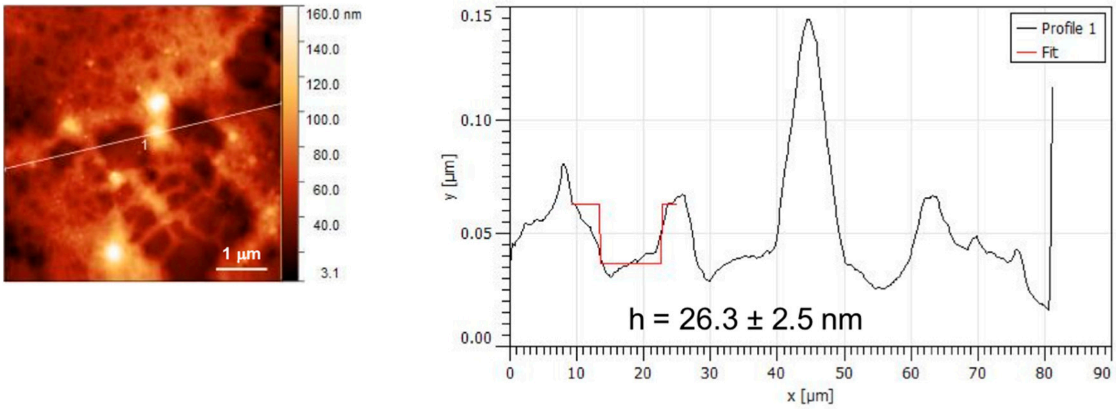

S5c.

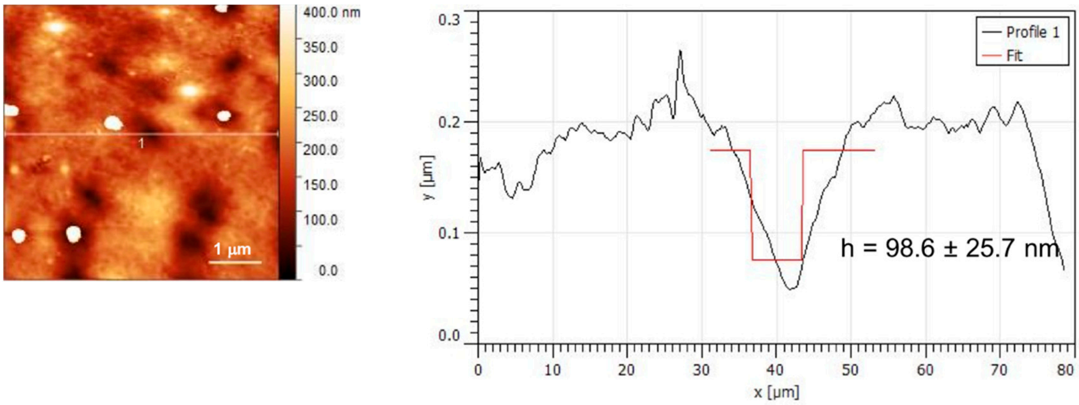

S5d.

Figure S5. Cross sections of the unbaked **(a)** and baked films at 140 °C **(b),** 150 °C **(c)** and 170 °C **(d)**. In each panel the AFM image of the film (on the left, consider Figure 4 in the manuscript) and the cross-section (right) measured along the line marked 1 in each AFM image is shown. In order to compare the morphology of each film, the depth of voids statistically present on the film surface are also given.

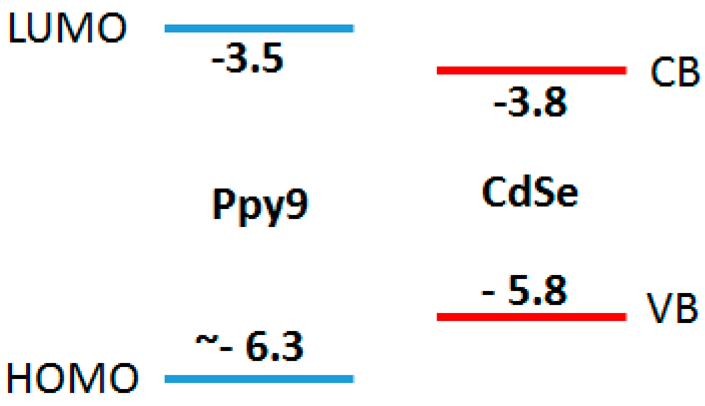

Figure S6. Energy levels of polymer Ppy9 and CdSe, showing type 1 alignment.
